# Ultra-small dye-doped silica nanoparticles via modified sol-gel technique

**DOI:** 10.1007/s11051-018-4227-1

**Published:** 2018-04-25

**Authors:** R. Riccò, S. Nizzero, E. Penna, A. Meneghello, E. Cretaio, F. Enrichi

**Affiliations:** 1 Institute of Physical and Theoretical Chemistry, Graz University of Technology, Stremayrgasse 9, 8010 Graz, Austria; 2 Department of Physics, University of Padova, via Marzolo 8, 35131 Padua, Italy; 3 Department of Biomedical Sciences, University of Padova, Via Ugo Bassi 58/B, 35131 Padua, Italy; 4 CRO National Cancer Institute, Via Franco Gallini 2, 33081 Aviano, PN Italy; 5 Department of Molecular Sciences and Nanosystems, University of Venice Ca’ Foscari, Via Torino 155, 30172 Mestre, VE Italy; 6 Division of Materials Science, Department of Engineering Sciences and Mathematics, Luleå University of Technology, 971 87 Luleå, Sweden; 7 Museo Storico della Fisica e Centro Studi e Ricerche Enrico Fermi, Piazza del Viminale 1, 00184 Roma, Italy

**Keywords:** Silica nanoparticles, Dye doping, Luminescence, Biosensing, Bioimaging applications

## Abstract

In modern biosensing and imaging, fluorescence-based methods constitute the most diffused approach to achieve optimal detection of analytes, both in solution and on the single-particle level. Despite the huge progresses made in recent decades in the development of plasmonic biosensors and label-free sensing techniques, fluorescent molecules remain the most commonly used contrast agents to date for commercial imaging and detection methods. However, they exhibit low stability, can be difficult to functionalise, and often result in a low signal-to-noise ratio. Thus, embedding fluorescent probes into robust and bio-compatible materials, such as silica nanoparticles, can substantially enhance the detection limit and dramatically increase the sensitivity. In this work, ultra-small fluorescent silica nanoparticles (NPs) for optical biosensing applications were doped with a fluorescent dye, using simple water-based sol-gel approaches based on the classical Stöber procedure. By systematically modulating reaction parameters, controllable size tuning of particle diameters as low as 10 nm was achieved. Particles morphology and optical response were evaluated showing a possible single-molecule behaviour, without employing microemulsion methods to achieve similar results.

**Graphical abstract:**
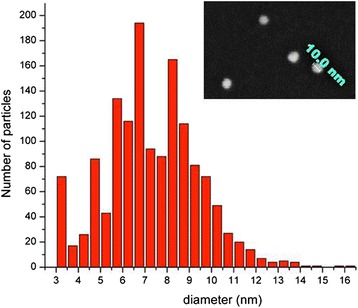
We report a simple, cheap, reliable protocol for the synthesis and systematic tuning of ultra-small (< 10 nm) dye-doped luminescent silica nanoparticles.

We report a simple, cheap, reliable protocol for the synthesis and systematic tuning of ultra-small (< 10 nm) dye-doped luminescent silica nanoparticles.

## Introduction

A fundamental requirement for highly responsive optics-based biosensors is to have enough signal-to-noise ratio (SNR) to reveal the presence of analytes at very low concentrations (Banica 2012). Huge efforts were spent along the years to develop stable, efficient, highly emitting optical labels with optimal spectral properties, with cost-effective production procedures easy to scale up for commercial production (Syahir et al. 2015). Among these properties, the most important ones required for optical labels for biosensing technologies are high biocompatibility, high SNR, and low photodegradation. In an initial effort of developing alternative strategies for high sensitivity detection using time-delayed analysis, the use of Europium-doped silica NPs was found to provide enough long-living signal (in the order of milliseconds) from the emission of rare earth ion with respect to the short-living self-fluorescence (in the order of ns) of the biological system (Enrichi et al. 2008; Canton et al. 2011). Other rare earth metals, and luminescent silica NPs in place of directly link emitting molecules, were also explored (Enrichi 2008). One of the advantages of dye-doped nanoparticles over other luminescent nanoparticles like quantum dots or rare earth-doped NPs is to provide the same optical properties of the incorporated dyes, which are usually commercial dyes. Therefore, they are perfectly matched to the typical spectral response of standard laboratory instruments fitted to commercial dyes, but at the same time they can provide much higher performances. Indeed, the use of dye-doped silica NPs was demonstrated to improve significantly the detection limit of a microarray device (Enrichi et al. 2010), permitting the implementation of this fluorescent nanosystem in the in vitro DNA microarray detection of human papilloma virus (HPV) single-strand sequences (Riccò et al. 2011). All these studies suggest the use of *d*ye-*d*oped *si*licon *o*xide *n*ano*p*article*s* (ddSiONPs) as a promising strategy to overcome the drawbacks of fluorescent molecule labels. In fact, the surface of silica NPs can be easily modified to link any suitable bio-probe (Li et al. 2012). Furthermore, by incorporating dyes into silica spheres, a higher concentration of fluorescence emitters can be obtained within a single optical label, which yields to improve SNR, and the molecules inside the silica network are protected from the environment, reducing photobleaching (Canton et al. 2011). Small size is a key requirement for this new class of fluorescent labels, to ensure the system can be easily substituted to traditional single-molecule labels, to avoid photobleaching and photoblinking, and to improve chemical stability and bio-conjugation efficiency. The use of silica particles has also been shown very promising for clinical use, thanks to its high biocompatibility (Asefa and Zhimin 2012). The < 10-nm size is particularly important for in vivo applications, where the size is a major determinant in biodistribution and pharmacokinetic.

Low-size particles are often obtained using microemulsion methods (Esquena et al. 1997; Lade et al. 2000; Bagwe et al. 2004; Finnie et al. 2007). Several groups around the world have focused on developing methods in this field to fabricate ultra-small dye-doped silica particles with regular pore sizes. For example, Ma et al. systematically investigated the fabrication of both dense and mesoporous sub-10-nm silica particles, using CTAB surfactant and PEG-silane (Ma et al. 2012a, b). Recently, Quan et al., fabricated sub-10-nm fluorescent particles in alkaline buffer and in presence of cyclohexane to produce a biphasic system (Quan et al. 2017).

However, these techniques may present environmental and technical drawbacks hampering their scalability and making the production process less attractive for commercial applications. For example, the reaction vessels must be able to withstand organic compounds (solvents, surfactants) used for the inevitable water-in-oil emulsion, and base catalysts; some organic solvents can pose an environmental and health hazard; the surfactant must be chosen to precisely generate defined micelles; the doping agent must be hydrophilic to enter the inner aqueous micellar environment; and the work-up requires breaking of micelles and extensive washing to remove surfactant, solvents, oligomers, and other by-products.

To the purpose of biosensing, porosity is not a key factor, whether scalability is crucial to allow commercial applications. A classic and easily scalable Stöber method (Stöber et al. 1968) can provide mono-dispersed and well-defined particles, but only in the 100–1000-nm range, where “nano” systems become “micro” systems (Alemán et al. 2007). A notable reduction in NP size has been recently achieved (Canton et al. 2011) with a time- and cost-efficient technique following a modified Stöber basic environment sol-gel procedure, using tetraethyl orthosilicate (TEOS) and aminopropyl triethoxysilane (APTES) in ethanol environment, AlexaFluor® dyes as doping agents dissolved in dimethyl sulfoxide (DMSO), and ammonia solution as catalyst. NPs in the 15–70-nm range were easily produced, and the effect of dye loading and APTES amount in the resulting diameters was thoroughly studied (Canton et al. 2011).

In the present work, we will provide guidelines to synthesise nanosized luminescent silica labels for fluorescence-based biological sensing applications, showing a simple, efficient, and sustainable protocol for the synthesis and systematic tuning of ddSiONPs in the ultra-small scale range (< 30 nm), by modification of the classic base-catalysed, sol-gel hydrolysis, and condensation of TEOS (Brinker and Scherer 1990), using fluorescein as probe and, importantly, with analogous results that were obtained with microemulsion methods.

## Experimental

### Materials and methods

Tetraethyl orthosilicate (TEOS 98%), 3-aminopropyl triethoxysilane (APTES), 5(6)-carboxyfluorescein (FCOOH), dicyclohexylcarbodiimide (DCC), 4-(*N*,*N*-dimethylamino)-pyridine (DMAP), and ammonia solution (25 or 30% aqueous) were purchased from Sigma Aldrich.

Acetone, ethanol (EtOH), dimethyl sulfoxide (DMSO), ethyl acetate (EtOAc), and tetrahydrofuran (THF) were used without further purification.

SEM measurements were performed with a TESCAN Vega TS 5130 LM working at 30 kV. High-performance FESEM investigations were conducted on a dual-beam FEI Nova 600i instrument, with a semi-in-lens cold cathode field emission scanning electron microscope source. The micrographs were taken at 5-kV accelerating voltage, using in-lens detector in pure secondary electron signal mode. In all cases, a thin layer of gold was sputtered onto samples.

Atomic force microscopy (AFM) maps were produced using a NT-MDT Ntegra instrument in a semi-contact mode with gold-coated Si cantilevers Etalon or NSG01, 03, 10, 30 types. A typical measure scans a 75 × 75 to 5 × 5 μm^2^ area, with a scanning frequency between 0.2 and 0.5 Hz, depending on the surface.

DLS measurements were performed with a Malvern Instruments Zetasizer.

### Synthesis of dye adduct fluorescein APTES

In a round-bottom, two-neck, 250-mL Schlenck-lined flask, under magnetic stirring and nitrogen stream, 564 mg (1.5 mmol) of FCOOH is dissolved in 30 mL of anhydrous acetone. Two solutions of 402 mg (2 mmol) of DCC in 10 mL of acetone and 488 mg (4 mmol) of DMAP in 10 mL of acetone were added and the mixture was stirred for 15 min. Subsequently, 432 mg (463 μL, 2 mmol) of APTES was injected and the stirring continued for another 90 min.

After solvent removal, the residue was taken with EtOAc, washed 2 × 100 mL with citric acid (1% aqueous solution) and 1 × 100 mL with brine. Organic phase was dried over MgSO_4_ and solvent removed, leaving FCPTES as yellow solid (800 mg, 92%). TLC (SiO2, EtOAc/EtOH 8:2): *R*_f_ = 0.32. 1H-NMR (CDCl3, 300 MHz): 10.17 ppm (s, 1H, COOH), 7.19 ÷ 8.45 ppm (m, 7H, CH Ar xanthene + NH), 6.50 ÷ 6.75 ppm (m, 3H, CH phenyl), 4.03 ppm (t, 2H, N-CH_2_, *J* = 7.1 Hz), 1.58 ÷ 1.72 ppm (m, 10H, CH_2_ propyl and Si-O-CH_2_), 1.01 ÷ 1.25 (m, 9H, CH_3_). FTIR (KBr) 3479, 3329, 3061, 2929, 2851, 1749, 1629, 1510, 1384, 1244, 1184, 1111, 844, and 661 cm^−1^. UV-Vis (EtOH, 0.4 mM): *λ*_max_ = 454 and 484 nm.

### General methods for low-size (< 30 nm) silica nanoparticles synthesis

In a plastic, conical bottom, 50-mL vial provided with magnetic stirrer, ethanol, 18.3 MΩ deionised water, TEOS, and ammonia solution were added and mixed for 18–24 h. After that, purification method was followed as explained in the following section. For dye-doped particles, the fluorophore is previously dispersed in 2 mL of DMSO or ethanol (see synthesis details in Table 1). Fluorescein@SiO_2_ NPs were prepared using 10 mM ethanol or DMSO solutions of FCPTES adducts.

**Table 1:** Molar ratios of reaction species (TEOS = 1) with diameters obtained from dimensional measurements

Sample	EtOH	H_2_O	NH_3_	DMSO	FCPTES (× 10^−4^)	Avg. Ø (nm)
D0F2	86	33.2	2.4		2	32 ± 3^b^
D0F5	86	33.2	2.4		5	35 ± 3^b^
D14F2	86	33.2	2.4	14.2	2	3 ± 1^b^
D7F0	86	33.2	2.4	7.1		5 ± 1^b^ (14 ± 7^d^)
D3F0	86	33.2	2.4	3.5		15 ± 3^b^ (20 ± 7^d^)
E86	86	5.3	2.4	14.2	2	9 ± 2^c^
E129	129	5.3	2.4	14.2	2	13 ± 4^c^
E172	172	5.3	2.4	14.2	2	18 ± 5^c^
E215	215	5.3	2.4	14.2	2	8 ± 2^c^
E258	258	5.3	2.4	14.2	2	7 ± 2^c^
W2N1	200	2.2 (33.6^a^)	1		1	12 ± 4^c^
W4N2	200	4.4 (35.8^a^)	2		1	23 ± 5^c^
W8N4	200	8.8 (40.2^a^)	4		1	55 ± 8^c^
W17N8	200	17.6 (49.0^a^)	8		1	200 ± 20^c^
W26N12	200	26.3 (57.7^a^)	12		1	250 ± 25^c^

D7F0: PDI = 0.24; D3F0: PDI = 0.14

^a^Additional H_2_O = 31.4

^b^AFM

^c^SEM

^d^DLS

### General method for low-size (< 30 nm) silica nanoparticles purification

Amicon-15 ultrafiltration 50-mL vials (Millipore), with a 20-to 100-kDa cut-off, were used. The reaction mixture was diluted with the same volume of 18.3 MΩ deionised water, then poured in 10-mL portions in the upper part of the vial; every portion is centrifuged at 5000 rpm for 20 min, the underlying filtrate discarded, and the above concentrate dispersion collected. After completion, all the fractions were put together, diluted to 10 mL with 18.3 MΩ deionised water, put into an Amicon-15 vials (Millipore) with 50-kDa cut-off, and centrifuged at 5000 rpm for 30 min, with 2 × 25 mL water rinsing. Before the centrifugation step, vial was vigorously shaken to prevent filter clogging. The obtained concentrate solution is collected, diluted to 2 mL with water or buffer solution and stored at 4 °C.

## Results and discussion

**Fig. 1:**
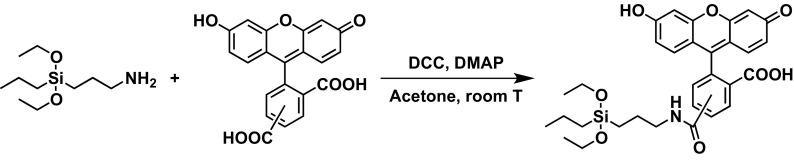
Reaction scheme for the synthesis of FCPTES. APTES reacts with 5(6) carboxyfluorescein to form FCPTES. Room *T* = 24 °C

The integration of fluorescein isothiocyanate (FITC) within inorganic silica frameworks is usually conducted by the addition APTES to FITC (Enrichi et al. 2009a, 2010; Riccò et al. 2011). Here, we explored an alternative pathway in order to extend this approach to other fluorescent molecules lacking the iso(thio)cyanate moiety. In this regard, we used a traditional amide formation method (Neises and Steglich 1978) for the synthesis of siloxane-derivatized dyes through the formation of an activated ester, using APTES and 5(6)-carboxyfluorescein, as depicted in Fig. 1.

This approach brings several advantages: fast reaction at room temperature thanks to *N*,*N*-dimethylaminopyridine (DMAP) acceleration, strong water harvesting by dicyclohexylcarbodiimide (DCC) to avoid siloxane hydrolysis, selective functionalization on the desired moiety, easy execution, high yield (> 90%), and environmental compatibility. The sol-gel silica nanoparticle synthesis has been performed according to the procedure pioneered by Stöber, Bogush, and van Blaaderen (Stöber et al. 1968; Bogush et al. 1988; van Blaaderen and Vrij 1992). In their work, TEOS in an ethanol solution along with variable amounts of ammonia and water underwent hydrolysis and condensation phenomena to produce small silicon dioxide spheres with different diameters, usually bigger than 30 nm.

In the first set of experiments, the chosen co-solvent (DMSO) was added to solubilise the dye in the reaction medium. Previous studies demonstrated the formation of two distinct families of nanoparticles with 400- and 750-nm diameter, a phenomenon credited to the DMSO presence. However, according to scanning near-field optical microscopy (SNOM) measurements, only 750-nm particles were found to contain dye molecules (Enrichi et al. 2009b). Here, the experiments were conducted varying the volumes of DMSO solution (Table 1, samples D14F2, D7F0, and D3F0). The typical molar ratio of sol-gel solutions TEOS/EtOH/H_2_O/NH_3_ was 1:86:33.2:2.4, corresponding to a mixture of 900 μL TEOS, 20 ml EtOH, 2.5 ml H_2_O, and 800 μL of 25% aqueous ammonia solution. Control samples were generated omitting DMSO (Table 1, samples D0F2 and D0F5).

In the second set of experiments, the concentration of reactants in solvent (EtOH) was modulated by varying the total amount of EtOH. This set (Table 1, samples E86 to E258) was produced fixing the TEOS/H_2_O/NH_3_/DMSO/FCPTES in the 1:5.3:2.4:14.2:2 × 10^−4^ M ratio, corresponding to 450 μL TEOS, 300 μL of 30% aqueous ammonia solution, and 2 mL of a FCPTES solution in DMSO (1 mg/mL). Ethanol was tuned from 86 to 258 equivalents with respect to TEOS, which resulted in a variable volume from 10 to 30 mL. In the third set of experiments, the investigation focused on the proportion between reactants and ammonia. In this final set (Table 1, samples W1N1 to W26N12), DMSO was not added, and a fixed TEOS/EtOH/H_2_O/FCPTES ratio 1:200:31.4:8.5 × 10^−5^ was used, corresponding to 450 μL TEOS, 23 mL EtOH, 1.125 mL H_2_O, and 0.1 mg of FCPTES. In this case, the ammonia molar amount rose from 33 to 58 equivalents, thus from 125 μL to 1.5 mL of 30% aqueous ammonia solution. The amount of water present as the diluent within the ammonia solution was included in the calculation of the total water amount reported in Table 1.

Nanoparticles with mean diameter of 32 ± 3 nm were measured for sample D0F2, whereas sample D0F5 provided particles with 35 ± 3 nm average diameter: the slight difference was attributed to the bigger amount of FCPTES used, that is, likely to have caused a slight rise in the average diameter due to the additional silica precursor added. When DMSO (2 mL) was added to the same solution, a significant diameter decrease was observed. The obtained nanoparticles passed from 32 ± 3 nm (sample D0F2) to 3 ± 1 nm (sample D14F2), a tenfold shrinking currently unreported in literature. Samples D7F0 and D3F0 were produced without dye doping, to investigate the influence of DMSO concentration on the mean diameter of the obtained nanoparticles. The dimensional trend with lowering DMSO concentration went from 5 ± 1 nm for D7F0 to 15 ± 3 nm for D3F0. AFM measurements were compared to dynamic light scattering (DLS) measurements: size trend as a function of DMSO concentration was consistent between the two methods. However, DLS measurements give an estimate of the hydrodynamic radius, whereas AFM measures the geometrical radius. Thus, DLS estimates of radii were systematically slightly higher as compared to those in AFM.

**Fig. 2:**
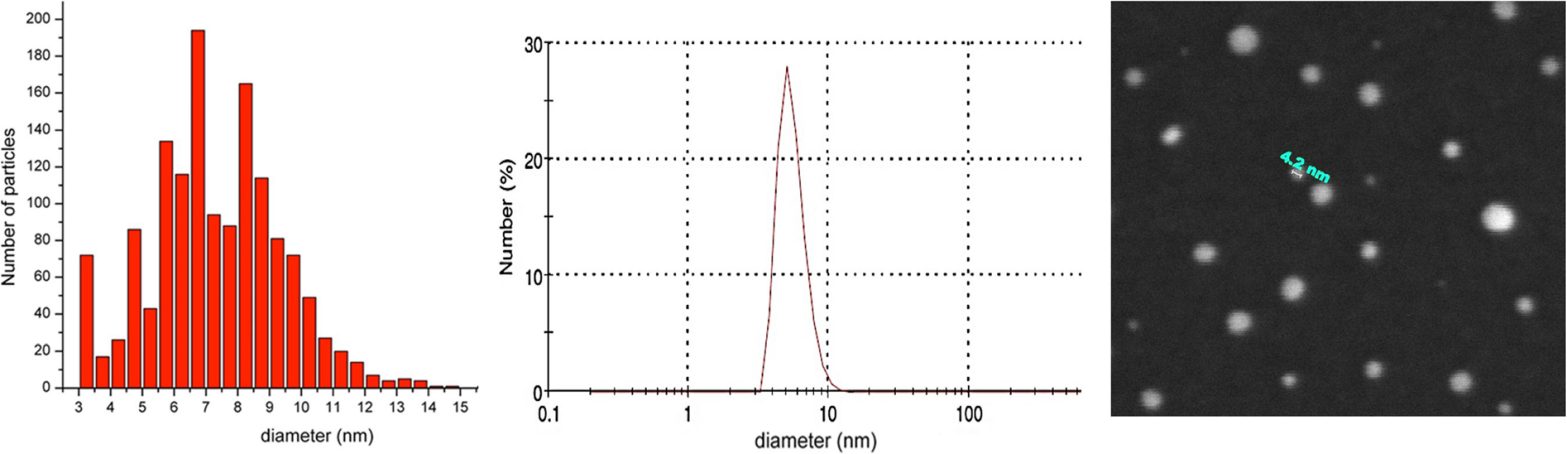
Size characterisation of samples E258 by AFM (left) and DLS (center). On the right, FESEM image of the same sample

The second series (E86-E258) has been produced removing the initial water and increasing the EtOH amount, to investigate the influence in the diameter. Sample size was investigated using AFM (Fig. 2 left), field emission scanning electron microscopy (FESEM, Fig. 2 right), dynamic light scattering (DLS, Fig. 2 center), and transmission electron microscopy (TEM). The obtained particles had actually an irregular distribution and seemed not to be heavily affected by the dilution. For example, microscopy measurements on sample E258 yielded an average diameter of 7 ± 2 nm. All the samples of the same set range between 7 and 18 nm, without a significant trend. From this investigation, it is clear that ethanol has a minor role in this range of size values. Instead, in this low diameter regime, the size is mainly determined by DMSO. Furthermore, by doubling the solvent molar ratio toward TEOS and drastically reducing the water amount, small particles under 20 nm were produced in presence of DMSO co-solvent. The mechanism behind this behaviour is likely due to the role of the polar aprotic solvent DMSO. It is well known that such class of solvents, albeit not hydrogen bonding, stabilises the reactants with respect to the activated complex, slowing down the reaction and inducing the formation of smaller nuclei (Brinker and Scherer 1990).

**Fig. 3:**
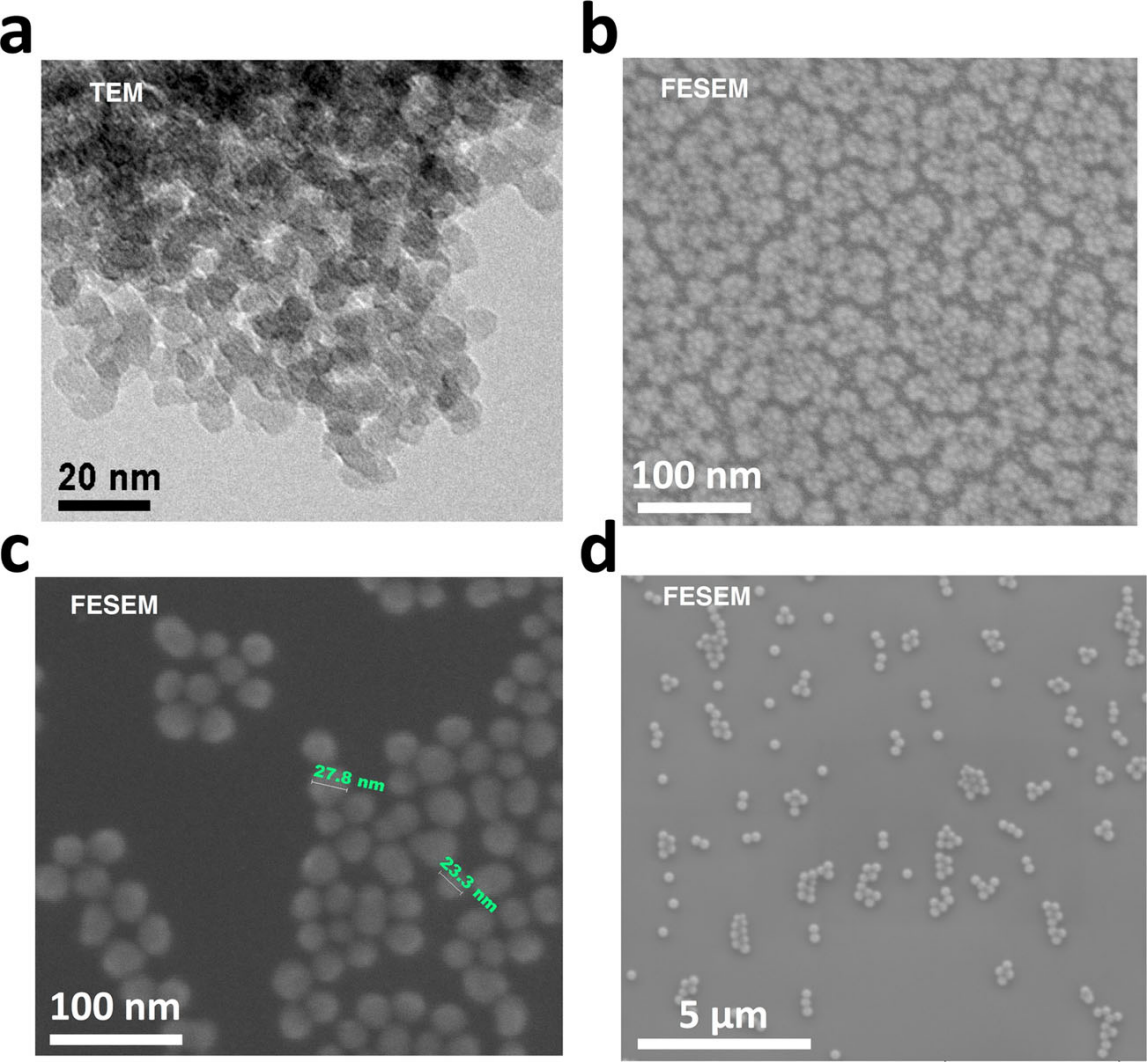
Micrographs for samples W2N1 (**a**), W4N2 (**b**), W8N4 (**c**), and W26N12 (**d**).

To achieve the goal of fine-tuning nanoparticle diameters in the range of ultra-small particles, the third set W2N1-W26N12 focused on using various ammonia concentrations. DMSO was removed and the ammonia molar ratio was tuned between 1 and 12, using a fixed TEOS/EtOH/H_2_O ratio of 1:200:31.4. FCPTES was kept at a 0.01% mol with respect to TEOS in all cases. It is noteworthy that proportions between ammonia, water, solvent, and other species are fundamental when producing silica nanoparticles by means of microemulsion systems (Bagwe et al. 2004). As seen in Fig. 3, nanoparticle dimensions were directly proportional to the amount of ammonia used, which can be explained based on sol-gel techniques established mechanism (Brinker and Scherer 1990.

**Fig. 4:**
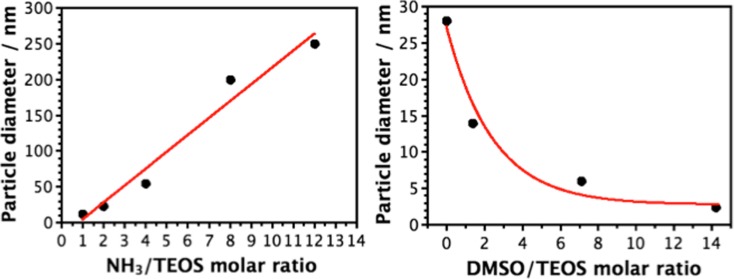
Dependence of particle size from amount of base catalyst (NH_3_ solution) (**a**) and amount of co-solvent (DMSO) (**b**)

The dimensional control was well maintained and it was possible to reach very low particle size by simply tuning the amount of base catalyst or co-solvent (Fig. 4). For samples W2N1 and W4N2, 125 and 250 μL of 30% aqueous ammonia solution were used, respectively. The aqueous molar ratios became 33 and 35 and the ammonia molar ratios were between 1 and 2 with respect to TEOS. Consequently, an equimolar amount of TEOS and ammonia was sufficient to produce very low sized silica nanoparticles, around 10 nm, in ethanol environment with nearly 15% of water. A twofold amount of ammonia was able to double the diameter without particular issues, thus providing a linear dependence between catalyst and dimension in the range 5–60 nm (samples W2N1, W4N2, and W8N4).

**Fig. 5:**
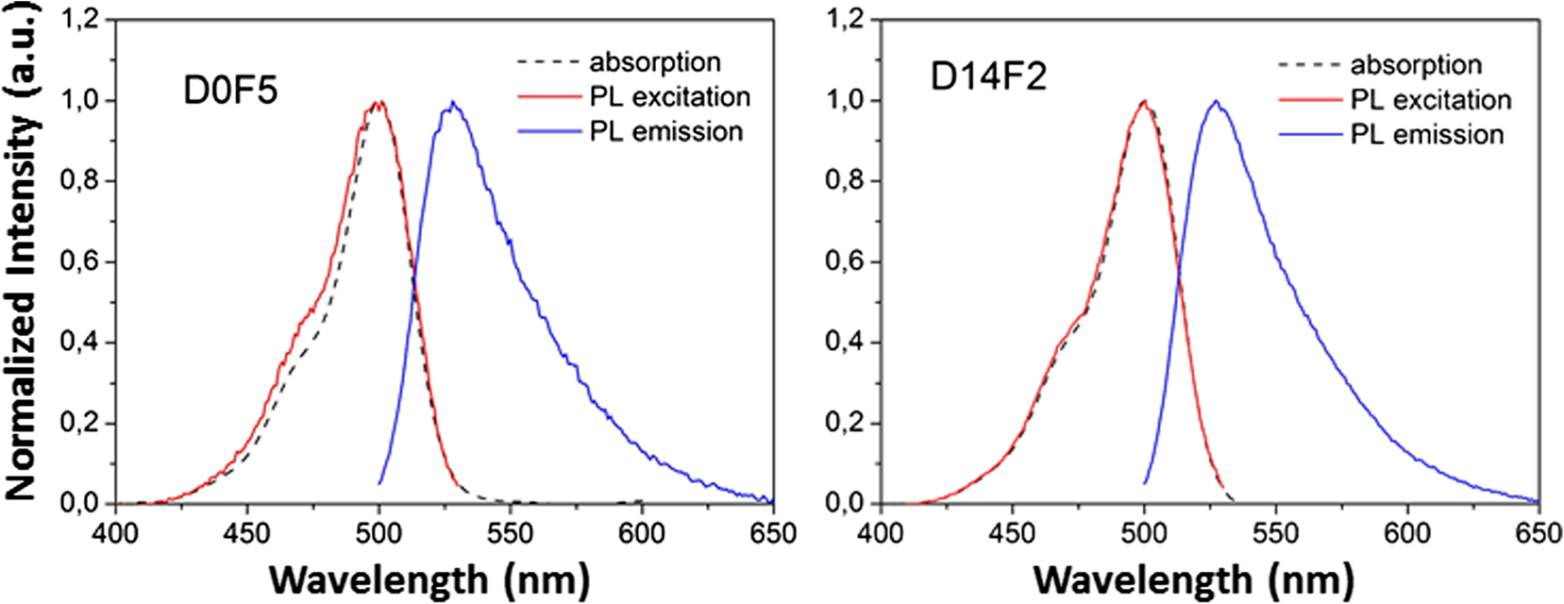
Absorption, PL excitation, and PL emission for the 35 ± 3 nm D0F5 (left) and 3 ± 1 nm D14F2 (right) nanoparticles. The PL excitation curve (red) was obtained monitoring emission wavelength *λ*_em_ = 530 nm. The PL emission curve (blue) was obtained under excitation wavelength *λ*_ex_ = 470 nm

Optical characterisation of the prepared nanoparticles confirmed the dye loading efficiency. Fluorescein was chosen as a widespread, cost-effective, and well characterised dye. In Fig. 5 left, the normalised optical absorption (black), photoluminescence (PL) excitation (red), and emission (blue) spectra of the 35 ± 3 nm D0F5 nanoparticles sample are reported. The clear difference between the PL excitation and the absorption spectra is attributed to the higher dye concentration in the synthesis and to the bigger particle size, allowing for the incorporation of dye molecules in close proximity, favouring energy transfer phenomena among them, and the probability of re-absorption or quenching phenomena. In Fig. 5 right, the normalised optical absorption (black), PL excitation (red), and emission (blue) spectra of the 3 ± 1 nm D14F2 nanoparticles are reported. These are in perfect agreement with the optical properties of a free dye, indicating incorporation of fluorescein in the nanoparticle without modification of its molecular structure (Lakowicz 2006). Moreover, the overlap between the photoluminescence excitation curve and absorption spectrum curve is complete and is indicative of no re-absorption or concentration-quenching processes. Thus, no deformation of the photoluminescence spectral shape both in the excitation and in the emission occurred. Given the reduced size of this sample and being a fluorescein molecule approximately 0.6 × 0.9 × 0.9 nm wide, it is reasonable to imagine either a single-molecule occupancy per particle or a single-molecule behaviour within a multiple-occupied particle, similarly to what has been previously demonstrated by Douhal and co-workers with another cyanine-based dye (Cohen et al. 2012). Future studies, beyond the scope of this work, would be necessary to assess the number of molecules per particle and to investigate the applicability of such systems to commercial biosensors.

## Conclusions

In this work, fluorescein-doped silica nanoparticles with a diameter down to 10 nm or less could be easily produced in an environmentally friend fashion, by a simple modification of the classical Stöber technique without the needs of microemulsion systems, avoiding the use of organic solvents and surfactants. This was achieved by adapting the amounts of ethanol and ammonia solution, reducing the amount of water. DMSO presence was found to induce the formation of very low-sized nanoparticles, but a single solvent would be preferred for the scalability of the production process. The technique of choice comprised a mixture of TEOS/EtOH/NH_3_/H_2_O in the molar ratio 1:200:1÷2:35, and can produce nanoparticles in the 10- to 20-nm range, as seen by AFM, DLS, and FESEM measurements. Dye doping was achieved by adding as low as 0.01% mol of dye with respect to TEOS. Furthermore, it could be easily adapted to other dyes, provided the possibility to their functionalization for the covalent bonding with the siloxane groups. This easy, time- and cost-efficient method to synthesise ultra-small fluorescent silica particles can open the way to the production of a new class of highly emitting luminescent markers for biosensing technologies. Finally, the procedure can be extended from a fluorescein-based dye to other fluorescent species for on-demand tuning of the optical properties.
